# The Use of Transdermal Buprenorphine for Acute Postherpetic Neuralgia: A Case Report

**DOI:** 10.7759/cureus.34886

**Published:** 2023-02-12

**Authors:** Theofilos Tsoleridis

**Affiliations:** 1 Anesthesia and Pain Treatment Unit, Rhodes General Hospital, Rhodes, GRC

**Keywords:** postherpetic neuralgia, transdermal buprenorphine, pain management, neuropathic pain, buprenorphine patch

## Abstract

The purpose of this study is to report a case in which standard medication protocols for postherpetic neuralgia (PHN) led to adverse effects and insufficient results. The dead end that occurred in association with the patient’s deteriorating general condition and hesitation to comply with oral medication due to fear led to the application of transdermal buprenorphine (TDB) after written informed consent was obtained. TDB application in acute cases is still under study, and the literature is limited.

A 78-year-old female presenting with intense PHN was treated with pregabalin and paracetamol but complained of inadequate pain control and intense somnolence. TDB 35 μg/hour was applied after written consent was given. Six hours later, the situation improved, while five days later, the patient recovered completely. The patch was removed on the sixth day after application. In the follow-up after two weeks, no neuropathic symptoms or adverse effects were reported.

The optimal results of TDB application were substantially represented by excellent and continuous pain control, improved quality of life, and patient compliance due to the pharmacological properties of buprenorphine and easy patch application. The patient’s rapid response to TDB’s soothing action is an encouraging factor for its application in studies regarding PHN and acute pain attacks in general.

## Introduction

Postherpetic neuralgia (PHN) occurs due to peripheral nerve damage after herpes zoster virus (HZV) reactivation and has typical neuropathic pain characteristics (allodynia and hyperalgesia). PHN is usually confined to a dermatome and is described as a continuous burning sensation or electric shock [1].

As referenced in the study by Tontodonati et al., PHN's most widely accepted definition is persistent pain in HZV-associated areas three months after rash healing [1]. More recently, PHN has been defined as clinically relevant pain that persists 120 days after HZV onset [1].

Currently, no treatment can modify the course of the disease [1]. PHN is contained with anticonvulsants (e.g., gabapentin and pregabalin), tricyclic antidepressants, and paracetamol [2]. Local anesthetic (lidocaine) and capsaicin patches are also used [3,4]. Opioids are usually reserved for extreme circumstances due to abuse and addiction concerns, while nonsteroidal anti-inflammatory drugs are thought to be ineffective against PHN [5,6].

This study aims to report a case in which standard medication protocols for PHN led to adverse effects and insufficient results. The dead end that occurred in association with the patient’s deteriorating general condition and her hesitation to comply with oral medication due to fear led to the application of transdermal buprenorphine (TDB) after her written informed consent was obtained. TDB application in acute cases is still under study. The study adheres to the applicable Enhancing the Quality and Transparency of Health Research (EQUATOR) guidelines, and written informed consent was obtained from the patient for the publication of this case report.

## Case presentation

The patient, a 78-year-old female, visited the Rhodes General Hospital, Rhodes, Greece, complaining of intense thoracic pain (visual analog scale = 10) located on the right chest side with thoracic 5 and thoracic 6 dermatomal distribution. She described an insidious and continuous burning sensation that manifested after the herpetic rash’s disappearance (with onset two months before) with the same segmental distribution. No other family members were affected by PHN.

The patient’s medical history revealed hypertension that was treated with olmesartan 25 mg and nebivolol 5 mg and depression treated with citalopram 20 mg and alprazolam 1 mg. She reported falling from a staircase three months earlier, resulting in a fracture of the right sixth, seventh, and eighth ribs. The fractured ribs’ pain was treated with etoricoxib 90 mg and paracetamol 1 g, which the patient reportedly took as needed. She considered that the stress due to the situation was responsible for the PHN, as it was the first and only incident.

Clinical examination revealed brownish hyperpigmentation of the affected area, typical of cicatricial skin evolution with hyperpathy, paresthesia, hyperalgesia, hypesthesia, and allodynia. Moreover, vast zones of hypoesthesia were present, alternating or overlapping the dysesthetic zones.

The patient was treated with pregabalin 75 mg and paracetamol 1 g and discharged. However, three days later, the pain persisted and further deteriorated her general condition. She also reported intense somnolence that prohibited her from completing her everyday tasks and was impatient about the medication used. Furthermore, she was anxious that her state would not improve. At this point, she was called to the hospital. The previous treatment was ceased, and a patch of TDB 35 μg/hour (the lowest dosage available in Greece) was applied every 72 hours. The patient was discharged and contacted by phone twice a day.

Six hours after the patch application, PHN appeared to be in regression, with constant and progressive improvement of the general condition without any collateral effects. Furthermore, no cutaneous reaction was noted, and the patient seemed to be encouraged and more optimistic, due to her sudden improvement.

Three days after patch application, no PHN paroxysmal attacks were noted, and no further pain relief medication was needed. The patient recovered her appetite and sleep. Five days after the patch application, she had recovered completely. Furthermore, the absence of pain and general improvement in sleep, appetite, and mood were noted. The patch was removed on the sixth day, and neither adverse effects nor pain was registered. No further patch application was required.

In the follow-up one month later, no neuropathic symptoms were present. The patient was notably satisfied and relieved with the use of the patch, as she completely distrusted and detested the oral medication. She has not yet presented other PHN incidents.

## Discussion

PHN pathophysiology is likely caused by sensorial nerve damage from HZV, leading to neurochemical, anatomical, and physiological modifications to both afferent and central neurons. As a result, sodium chloride (NaCl) channel accumulation brings hyperexcitability and downregulation of the tetrodotoxin-resistant voltage-gated sodium (Nav) 1.8 channel and upregulation of the tetrodotoxin-sensitive Nav1.3 and the transient receptor potential cation channel subfamily member V 1 channels [7].

These changes are crucial for the *N*-methyl-D-aspartate glutamate receptor-dependent excitability increase of the spinal dorsal horn neurons. Furthermore, magnetic resonance imaging has revealed brain stem and cervical cord lesions attributable to HZV in 56% of PHN patients. Risk factors for patients developing PHN include prodromal pain signs that appear with the rash, pain, and rash severity and age [7].

Buprenorphine represents a thebaine’s semisynthetic derivate, with a potency 25-40 times greater than morphine. It is a μ receptor’s strong partial agonist (in a low dosage) and κ, δ, and ε receptors’ antagonist (in a high dosage). It presents an increased lipophilic affinity that, combined with its low molecular weight and high analgesic potency, affects the construction of an advanced transdermal system of continuous and controlled drug release in the active state. The latter is contained in a polymeric matrix that also acts as an adhesive and reaches clinically effective plasma concentrations within 24 hours that are maintained for 72 hours [8].

The rationale for treating this patient with buprenorphine was based on the fact that opioids suppress the central response to the nociceptor input. According to Smith, despite the lack of specific evidence that opioids are effective against neuropathic pain, some opioids can contain it remarkably well individually [9]. These two facts, in association with the patient’s characteristics, led to the TDB application.

According to the literature, TDB use against PHN (and noncancer pain in general) remains inconclusive. In a study by Bruckenthal and Barkin, opioid use was defined as a second-line therapy against PHN. Nevertheless, TDB and buprenorphine in general were not reported [10]. In the studies by Kusnik et al., TDB was suggested to be valuable against chronic noncancer pain; however, they did not report any application in PHN cases [11,12]. To complicate things further, another large-scale study by Wiffen et al. suggested that there was no substantial evidence to support or dismiss buprenorphine’s efficiency against neuropathic pain, and the authors proposed conducting new clinical trials [13].

TDB was preferred instead of a pure opioid agonist, such as fentanyl, because, according to the studies by Wolff et al. and Arshad et al., TDB is more cost-effective and offers a longer duration of analgesia as well as significantly fewer adverse effects [14,15]. Buprenorphine’s partial agonist profile leads to a ceiling effect regarding respiratory depression in contrast to pure agonists and could occur with a dosage over 32 mg, which is equal to 70 times the analgesic dose range. Therefore, respiratory depression by TDB is highly unlikely [14,15]. Buprenorphine has been linked to a dose-dependent prolongation of the QTc at the electrocardiogram (ECG). However, there is no evidence of buprenorphine association with *Torsade des Pointes* [16]. The patient was assessed by her attending cardiologist four months before the TDB application and was presented with a normal ECG. Finally, somnolence (a side effect that the patient in our case complained of from the previous treatment) is reported in only 0.8% of patients [8].

In a meta-analysis by Khaliq et al., topical lidocaine (TL) application against PHN led to pain reduction in comparison to a placebo [3]. Nevertheless, the authors concluded that there was insufficient evidence to suggest TL use as a first-line medication against PHN. Furthermore, while the authors believed that some patients could benefit from TL individually, they suggested the use of other medication with stronger evidence (e.g., gabapentin) [3]. Based on that, it was decided to avoid the risk of applying medication with no guaranteed results in favor of the aforementioned rationale.

Capsaicin patches have been registering strong results against PHN. However, in a study by Wallace and Pappagallo, 20% of the patients needed medication for treatment-associated discomfort that could last for five days [4]. At that point, it was decided to avoid the risk of exposing the patient to such discomfort, as it could deteriorate her psychological condition.

In summary, the switch from the previous treatment to TDB could be considered aggressive. However, considering the patient’s continuing physical and mental deterioration, her fear and hesitation to comply with oral medication regimens in association with her increasing distrust led to drastic measures. TDB was preferred to other opioids due to longer analgesia duration and fewer adverse effects (especially respiratory depression and somnolence) [14,15]. As both TL and opioids seem to offer remarkable results individually, TDB was preferred due to its central response suppression [3,9]. Moreover, thanks to TDB’s partial agonist profile, its full analgesic potency could be harnessed with no adverse effects [8]. In contrast, adverse effects could hinder a pure agonist’s full analgesic properties for the same analgesic result. Antidepressant medication was avoided as it could alter the medication regimen and the patient’s psychiatric condition^ ^[6]. Finally, TDB was preferred to capsaicin due to treatment-associated discomfort risk [4].

## Conclusions

The optimal results of TDB application were substantially represented by excellent and continuous pain control, improved quality of life, and the patient's compliance due to the aforementioned pharmacological properties of buprenorphine and easy patch application. The patient’s rapid response to TDB’s soothing action is an encouraging factor for its application in studies related to PHN and acute pain attacks in general.
